# Human Bocaviruses Are Not Significantly Associated with Gastroenteritis: Results of Retesting Archive DNA from a Case Control Study in the UK

**DOI:** 10.1371/journal.pone.0041346

**Published:** 2012-07-24

**Authors:** Sameena Nawaz, David J. Allen, Farah Aladin, Christopher Gallimore, Miren Iturriza-Gómara

**Affiliations:** Virus Reference Department, Health Protection Agency, London, United Kingdom; Centers for Disease Control and Prevention, United States of America

## Abstract

Gastroenteritis is a common illness causing considerable morbidity and mortality worldwide. Despite improvements in detection methods, a significant diagnostic gap still remains. Human bocavirus (HBoV)s, which are associated with respiratory infections, have also frequently been detected in stool samples in cases of gastroenteritis, and a tentative association between HBoVs, and in particular type-2 HBoVs, and gastroenteritis has previously been made. The aim of this study was to determine the role of HBoVs in gastroenteritis, using archived DNA samples from the case-control Infectious Intestinal Disease Study (IID). DNA extracted from stool samples from 2,256 cases and 2,124 controls were tested for the presence of HBoV DNA. All samples were screened in a real time PCR pan-HBoV assay, and positive samples were then tested in genotype 1 to 3-specific assays. HBoV was detected in 7.4% but no significantly different prevalence was observed between cases and controls. In the genotype-specific assays 106 of the 324 HBoV-positive samples were genotyped, with HBoV-1 predominantly found in controls whilst HBoV-2 was more frequently associated with cases of gastroenteritis (p<0.01). A significant proportion of HBoV positives could not be typed using the type specific assays, 67% of the total positives, and this was most likely due to low viral loads being present in the samples. However, the distribution of the untyped HBoV strains was no different between cases and controls. In conclusion, HBoVs, including HBoV-2 do not appear to be a significant cause of gastroenteritis in the UK population.

## Introduction

In 2005 a novel parvovirus was discovered in respiratory secretions of young children and was termed Human Bocavirus (HBoV-1) [1]. Other important members of the parvoviridae family include B19 which causes fith disease and human parvovirus 4 (Parv 4) which has not yet been associated with a disease [2]. Parvoviruses in animals are generally associated with systemic disease but also with respiratory and enteric symptoms [3], [4]. Since the discovery of HBoV-1 three other HBoV genotypes have been described, HBoV-2, HBoV-3 and HBoV-4. The association between HBoV-1 and respiratory disease has previously been well established [5], [6], [7], [8], [9], [10], [11], [12], [13], [14], [15], [16], [17], [18], [19]. Although all HBoVs have also been detected in stool samples with prevalences ranging from <1% to 20%, only HBoV-2 has been reported to be associated with symptoms of gastroenteritis [20]. Nevertheless, the role of HBoV2 as an aetiological agent of gastroenteritis has not been clearly confirmed, furthermore, to date, no clear association between the presence of HBoV-3 and HBoV-4 and disease has been established [21], [22].

Recent seroepidemiological studies indicate that exposure to HBoVs occurs early in life and 90% of the population are seropositive by the age of 5, although differences were reported in the seroprevalence of type-specific antibodies to the different HBoVs, which suggested that HBoV-1 infections are more prevalent [23].

The Infectious Intestinal Disease Study (IID Study) was a large case control study of gastroenteritis carried out in the UK between 1993–1996 [24] with the aim to determine the burden and aetiology of sporadic cases IID in the UK population. Initially, the use of classical microbiology diagnostic methods and electron microscopy (for virus detection) failed to detect a potential aetiological agent or toxin in 49% of the cases [25]. Retesting of the archived samples from this study using molecular methods for the detection of enteric viruses, bacteria and protozoa revealed viruses to be the most common aetiological agents of gastroenteritis,the diagnostic gap for IID was reduced to 25% from 49% [26]. The aim of the present study was to evaluate the role of HBoVs in IID in the UK population, using archived DNA samples from the matched case-control IID-1 study [26]. In addition, the presence specifically of HBoV-1, 2 or 3 was investigated in order to determine any possible associations between specific HBoV genotypes and IID.

## Materials and Methods

### Samples

A total of 4,380 archived DNA from the IID study [26], [27] were tested for the presence of HBoV DNA. This archive comprised DNA extracted from stool samples from 2,256 cases and 2,124 controls.

### Pan- HBoV Detection Assay

The qPCR assay targeted the NS1 gene (Ratcliff et al., unpublished method, personal communication) and was performed using an ABI Taqman7500. Oligonucleotide primer and probe sequences and positions are described in table 1.

**Table 1:** HBoV-specific oligonucleotide primers and probes (all located at the NS gene).

Primers	Sequence (5′-3′)	Nt positions	reference
Pan qpcr primers			
Pan-HBoV-F	ATA AAG TTC CAA ACT CAT TTC CTC TTG	1994–2020	Ratcliff et al*
Pan-HBoV-R	AGT GCA GWA TCC GTT TTC GTG	2079-2059	Ratcliff et al*
Pan HBoV1-F	TCT CC GGC GAG TGA ACA TC	201–219	This study
Type-specific qpcr primers (anti-sense)			
HBoV1-R	CAT CCG GAT GAG GAG CGC	424-407	This study
HBoV2-R	CTT CAG GAT GTG GTG CGC	427-410	This study
HBoV3-R	CAT CCG GAT GAG GA CAC	405-392	This study
Generic PCR primers for sequencing			
HBoV01.2F	TAT GGC CAA GGC AAT CGT CCA AG	2091	[29]
HBoV02.2R	GCC GCG TGA ACA TGA GAA ACA GA	1791	[29]
Probes			
pan HBoV-NS1	6FAM-CCT TTG TCC TAC WCA TTC-MGBBNFQ	2025–2042	Ratcliff et al*
HBoV-1	6Fam- TAT CAT AGA TTG TTC AGT TCC AGT AGC-MGBBNFQ	393-372	This study
HBoV-2	6Vic- TT GGA TCA TGA GAC GTT CAG TCC C-MGBBNFQ	399-376	This study
HBoV-3	6Ned- CTG GAT CAT GGC TTG CTC GGT A-MGBBNFQ	377-351	This study

* Unpublished method, personal communication.

The reaction consisted of 0.1 M DDT (Invitrogen), 1X Platinum Quantitative PCR Supermix-UDG (Invitrogen), Pan-HBoV-F and Pan-HBoV-R primers each at a concentration of 100 µm, Pan-HBoV-NS1 probe at 10 µm concentration, ROX 25 µm (Invitrogen) 2.5 µl of template DNA and RNase free water to a final reaction volume of 25 µl. The amplification consisted of an initial denaturation at 95°C for 10 min, followed by 40 cycles with denaturation at 95°C for 15 sec, annealing at 55°C for 30 sec and extension at 60°C for 45 sec.

### HBoV1, 2 and 3 Genotyping qPCR Assays

HBoV-1, 2 and 3-specific primer pair and probes were designed in house through alignment of sequence data available in GenBank. The reaction conditions are as follows; 1X Platinum Quantitative PCR Supermix-UDG (Invitrogen), HBoV-NS1-1F, HBoV-1R, HBoV2-R and HBoV3R primers each at a concentration of 20 µm, the HBoV1,2 and 3 probe at 10 µm concentration, ROX 25 µm (Invitrogen), 2.5 µl of template and RNase free water was added to a final reaction volume of 25 µl. The amplification conditions for the typing assay are the same as those described in the HBoV NS1 detection assay above.

### Plasmid Controls

Plasmids containing a 1773 bp and a 1737 bp region of the NS1 encoding gene of HBoV-1 and HBoV-3 respectively were used for assay optimisation and as controls. Control material was kindly provided by R. Ratcliff, Adelaide, Australia.

The controls were also used in order to generate a standard curve for use with the pan-HBoV assay in order to allow for normalisation of the data generated including the comparison of relative sensitivities of the different assays and for quantitation of DNA present in each of the positive samples. The standard curve was generated using the plasmid containing a genome segment of the HBoV-1 and consisted of a series of 10 fold dilution containing from 300,000 copies/µl down to 3 copies/µl. Inter- and intra-assay reproducibility was analysed by performing replicate testing of the standards in a single run (X11) and repeated runs (X2), and the standard curve was also included in each assay run for quality control and normalisation of results.

### Untypable Strains

A subset of 17 samples positive in the Pan-HBoV assay but which failed to amplify in the type-specific assays were confirmed using an alternative method published elsewhere [28], [29], and 6 were further confirmed though direct sequencing of the amplicons obtained after purification either from solution or agarose gels using Agencourt AMPure (Beckman Coulter, USA) and GeneClean Spin kit (QBiogene), respectively, following manufactures protocols.

### Statistical Analysis

The chi-squared test was used in order to evaluate the significance of differences observed between groups. For comparison of median values (analysis of CT values) the Mann Witney U-test was used. Prevalence Odds Ratio (POR = Pcases/(1-Pcases)/Pcontrols/(1-Pcontrols)) was calculated in the total cohorts and by age group.

**Table 2:** Age distribution of HBoV positive samples in cases and controls of IID.

	CASES			CONTROLS				ALLTOTAL		
Age Group (years)	HBoV pos	%	TOTAL	HBoV pos	%	TOTAL	POR	HBoV pos	%	TOTAL
<1	34	26.2	130	62	34.8	178	0.66514	96	31.2	308
1–4	58	12.2	476	91	18.0	506	0.633	149	15.2	982
5–9	9	6.9	131	8	6.0	134	1.16112	17	6.4	265
10–19	5	4.4	114	2	1.9	103	2.37635	7	3.2	217
20–29	7	2.4	286	2	1.1	177	2.21088	9	1.9	463
30–39	8	2.2	365	3	1.0	294	2.22699	11	1.7	659
40–49	11	4.4	249	2	0.8	240	5.70711	13	2.7	489
50–59	8	4.0	199	1	0.5	192	8.29167	9	2.3	391
60–69	5	2.9	175	3	1.7	177	1.72696	8	2.3	352
>70	4	3.1	131	1	0.8	123	3.96698	5	2.0	254
**TOTAL**	**149**	**6.6**	**2256**	**175**	**8.2**	**2124**	**0.79109**	**324**	**7.4**	**4380**

POR = prevalence odds ratio.

## Results

### Prevalence of Infection with HBoVs

A total of 7.4% of the samples tested were positive for HBoV. No statistically significant differences were seen in the prevalence of HBoV between cases and asymptomatic controls, POR = 0.79 (Table 2). Peak HBoV infection was observed in children under the age of 5, both in cases and controls, with significantly higher HBoV incidence in children between 1 and 4 in asymptomatic controls than in the cases of gastroenteritis (POR = 0.6; p<0.02). The number of HBoV positives in older age groups was too small for meaningful statistical analysis.

### Viral Load

**Figure 1:**
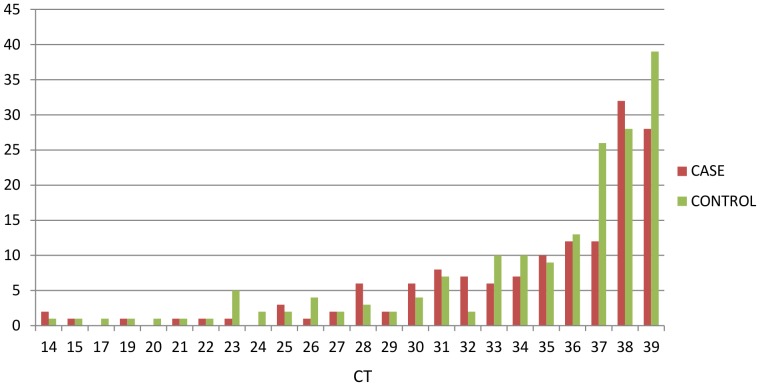
HBoV detection assays, CT distribution in cases and controls.

The average CT values were 34.5 and 34.8, and the median CT values were 36.3 and 37.4 in cases and controls respectively (see distribution in Figure 1). The majority of HBoV-positives in both cases and controls had copy numbers ranging between 30 and 299 copies/reaction (or between 4.5×10^3^ and 4.5×10^4^ copies/ml of feaces). The distribution of HBoV viral loads between cases and controls was comparable and the median CT values between cases and controls were not significantly different (U-test; z = 0.458139, p>0.05).

### HBoV in the Presence of Other Enteric Pathogens

HBoV DNA was found in 149 (46%) samples in the absence of other co-pathogens (Table 3). No statistically significant differences were observed in the proportion of cases or controls in which HBoV was found as a single organism or in the presence of one or more pathogens in the cohort as a whole, however in the 1–4 years of age group, HBoV in the absence of any other enteric pathogens was found in 29% of the cases, but in 56% of the controls (p<0.05).

**Table 3:** Distribution of HBoV-positive samples in cases and controls with or without a co-infection.

	CASES		CONTROLS		TOTAL	
Age Group	Single HBoV	Multiple infections	Single HBoV	Multiple infections	Single HBoV	All HBoV positives
<1	18	16	24	38	42	96
1–4	17	41	51	40	68	149
5–9	2	7	4	4	6	17
10–19	0	5	1	1	1	7
20–29	4	3	2	0	6	9
30–39	4	4	2	1	6	11
40–49	6	5	2	0	8	13
50–59	5	3	1	0	6	9
60–69	3	2	1	2	4	8
>70	2	2	0	1	2	5
TOTAL	61	88	88	87	149	324
**% of total positives**	**40.9**	**59.1**	**50.3**	**49.7**	**46.0**	

### Temporal Distribution of HBoV Infections

**Figure 2:**
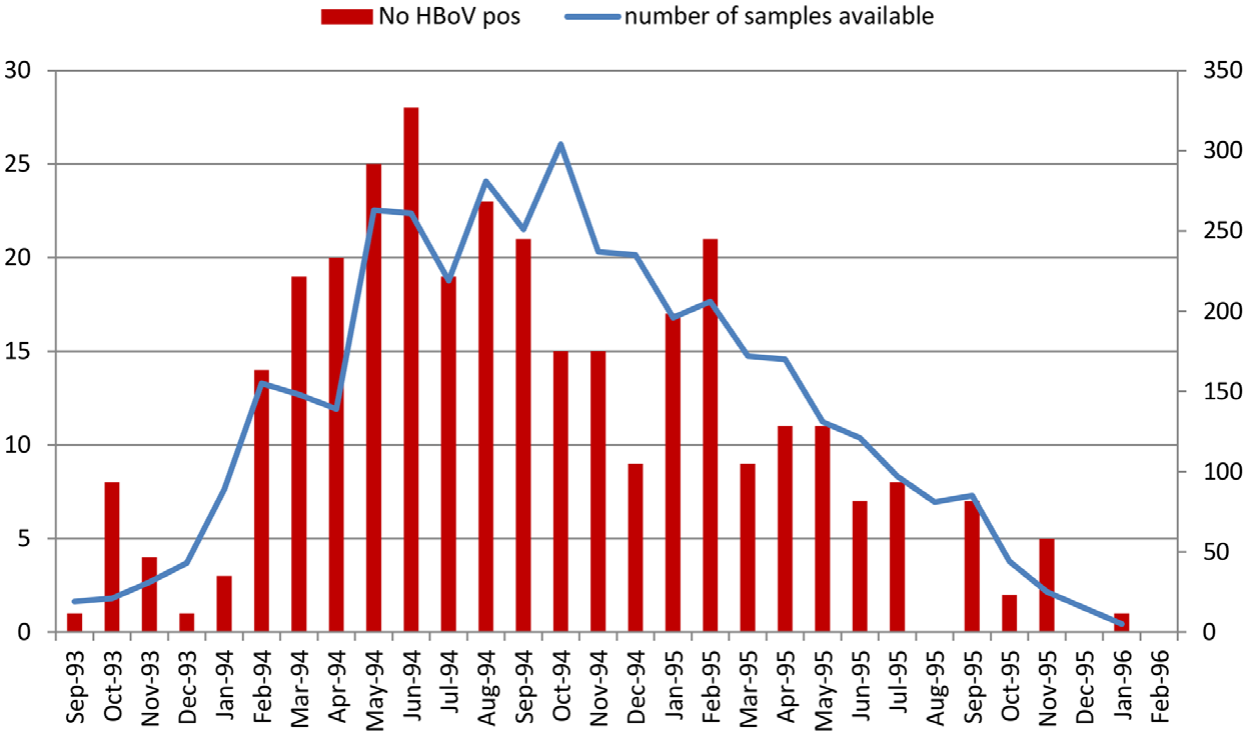
Temporal distribution of HBoV infections.

HBoV infections were detected year round although a peak was observed in the spring/early summer months, between April and June 1994 (Figure 2).

**Table 4:** Distribution of HBoV genotypes in cases and controls.

COHORT	Age Group(years)	HBoV-1	HBoV-1+HBoV-2	HBoV-2	HBoV-3	Untyped	TOTAL
CASES	<1	1	1	9	2	21	34
	1–4	3	1	8	6	40	58
	5–9			1	1	7	9
	10–19			2	1	2	5
	20–29			2		5	7
	30–39			3		5	8
	40–49			2		9	11
	50–59			3		5	8
	>70			2		2	4
	60–69					5	5
CASES Total		4	2	32	10	101	149
**Percent**		**2.7**	**1.3**	**21.5**	**6.7**	**67.8**	**100.0**
CONTROLS	<1	10	1	10	4	37	62
	1–4	14		8	6	63	91
	5–9				1	7	8
	10–19					2	2
	20–29					2	2
	30–39				2	1	3
	40–49					2	2
	50–59				1		1
	60–69			1		2	3
	>70					1	1
CONTROLS Total		24	1	19	14	117	175
**Percent**		**13.7**	**0.6**	**10.9**	**8.0**	**66.9**	**100.0**
**GRAND TOTAL**		**28**	**3**	**51**	**24**	**218**	**324**
**Percent**		**8.6**	**0.9**	**15.7**	**7.4**	**67.3**	**100.0**

### Distribution of HBoV by Gender

HBoV DNA was found in 48.8% and 45.7% of female cases and controls, respectively. The distribution of HBoV among females and males was not significantly different from the distribution of females and males in the entire cohort which was 53% and 47%, respectively.

### Distribution of HBoV Genotypes

A total of 106 (32.7%) HBoV positives were genotyped, whilst 218 (67.3%) remained untyped after testing in the HBoV types 1, 2 or 3 specific assays (Table 4). HBoV-1 detection was found predominantly in controls, (p<0.001) and HBoV-2 was predominantly associated with cases (p<0.01). The prevalence of HBoV-3 was not significantly different between cases and controls. HBoV-1 and -3 were predominantly found in children (Table 4). HBoV-2 in the absence of any other pathogen was detected in 17 (81.3%) of the cases, compared to 9 (47.4%) of the controls. In cases, HBoV-2 was found across the age groups, although more frequently in children <5, whereas in controls they were found predominantly in children <5 with only 1 example in an adult (Table 4). The prevalence of HBoV-2 in children <5 years old was however not significantly different between cases and controls, 2.8% and 2.6%, respectively.

A subset of HBoV that were negative in the type 1,2 or 3-specific assays were confirmed in an alternative pan-HBoV PCR, and sequencing of a small number confirmed them as types 1, 2 or 3. The majority of the untyped samples (70%) had a CT value of >37 in the screening pan-HBoV PCR, indicative of low viral loads being present in the samples.

## Discussion

This represents the largest study to date investigating the role and distribution of HBoVs infections in community acquired sporadic gastroenteritis and in asymptomatic controls. The prevalence of HBoV infection in the UK population was found to be 7.4% across all ages, with a higher percentage of the infections occurring in children <5 years of age (19%). However, the prevalence of HBoV infections was comparable in cases of gastroenteritis and in age-matched asymptomatic controls. Although the presence of enteric pathogens, eg norovirus or rotavirus, in asymptomatic individuals is well documented, a significantly higher prevalence of the pathogen is seen in cases than in the controls [26]. Therefore, our data suggests that HBoV are not causally associated with gastrointestinal disease in the UK population as a whole, nor in children. The prevalence of detection of HBoV in stool samples in previous studies varies widely (see summary in Table 5), but most coincide in reporting the highest prevalence in children.

**Table 5:** Summary of published studies on the prevalence of HBoV in stool samples.

Country	Sampling date	Cases No	Controls No	Population studied	% CasesHBoV-pos	% Controls HBoV-pos	Main Conclusion	Ref
Australia	Jan-Dec 2001.	186	186	Paediatric	17.20%	8.10%	HBoV-2 associated with gastroenteritis (only if cases with a concomitant bacterial infection included).	[22]
					(HBoV-2)	(HBoV-2)	Temporal pattern observed : summer months.	
Brazil	Jan 2003–Dec 2005.	705	ND	Paediatric	2.0% (HBoV)	ND	No obvious temporal clustering of the HBoV-positive patients.	[39]
China	July 2006–Sept 2007.	397	115	Paediatric	3.50% (HBoV)	3.50% (HBoV)	HBoV not associated with gastroenteritis. Temporal patterns observed : winter months.	[28].
China	July 2006–June 2008.	632	162	Paediatric	20.40% (HBoV-2)	12.30% (HBoV-2)	No statistically significant association between HBoV detection and gastroenteritis.	[32]
Germany	Jan-Feb and Sept–Dec 2007.	307	ND	Paediatric (daycare outbreaks)	4.60% (HBoV)		HBoV was not associated with outbreaks of gastroenteritis in childrenin day care.	[40]
Hong Kong	Nov 2004–Oct 2005.	1,435	ND	ND	2.10%		Same virus found in respiratory and stool samples.	[13]
Korea	Jan 2005–Dec 2006.	962	ND	Paediatric	0.80% (HBoV)		Temporal patterns observed : summer months.	[41]
South Korea	May 2008–April 2009.	358	ND	Paediatric	0.5% (HBoV-1) 3.6% (HBoV-2)		Temporal pattern observed : winter months.	[31]
Spain	Dec 2005–Mar 2006.	527	ND	Paediatric	9.10% (HBoV)		Prevalence of HBoVs in respiratory and stool samples was similar.	[42]
Thailand	Nov 2005–Sep 2006.	225	202	Paediatric	0.90% (HBoV)	0	Low prevalence. No statistically significant difference between casesand controls (p = 0.17).	[43]
USA	Dec 1–Marc 31 2008.	479	ND	Paedriatic and adult	1.30% (HBoV2)		0.7% children and 1.5% adults.	[45]

HBoV infections were detected all year round in the UK although a tentative peak was observed in the spring/early summer months in 1994 (between April and June). Different seasonal patterns in the peak prevalence of HBoV have been reported in different countries (see Table 5),

Of the 324 HBoV positive samples, 106 (32.7%) were genotyped in the type-specific assays. HBoV-1 was found predominantly in controls (p<0.001) and the prevalence of HBoV-3 was similar in cases and controls. Both HBoV-1 and -3 were predominantly found in children. HBoV-2 was predominantly associated with gastroenteritis cases (p<0.01). The overall prevalence in cases was 1.4% and 0.8% in controls, however, in children <5 year of age, the prevalence in cases and controls was similar, 2.8% and 2.6%, respectively. The prevalence of HBoV-2 in children in the UK was significantly lower than that reported in a study in Australia, in which HBoV-2 was detected in 17.2% and 8.1% of the cases and controls, respectively [22]. The findings of the study in Australia lead to the proposal of HBoV-2 as an important aetiological agent of infantile gastroenteritis. It is noteworthy however, that in the Australian study, the association of HBoV-2 with gastroenteritis was only significant when cases with a bacterial co-pathogen were included in the analysis. Although in the present study HBoV-2 in the absence of other enteric pathogens was found more frequently in cases than in controls, the small numbers found in such large study suggest that the role of these viruses in IID, if any, is likely to be small. A lack of correlation between HBoVs or HBoV-2 and paediatric gastroenteritis was also reported in several smaller studies published elsewhere [30], [31], [32].

A total of 67% of the HBoV-positive samples could not be genotyped using the genotype-specific PCR assays. The majority of these untyped samples (70%) had CT values >37. This suggests that failure to type may be associated with low viral loads and differences in the relative sensitivities of the genotyping assays compared to the detection assay. Although under experimental conditions and using plasmid controls the sensitivities of all assays were comparable, it is likely that when applied to true clinical samples the sensitivity of the type-specific assays was inferior, possibly due to as yet not identified strain variability within genotypes. Also, a HBoV type 4-specific assay was not included in this study, therefore, any possible HBoV4 infections would not have been typed. Of the panel of samples that were tested in an alternative pan-HBoV PCR, the strains typed through sequencing were HBoV-1 (2 samples), HBoV-2 (1 sample) and HBoV-3 (3 samples). Furthermore, the distribution of untyped HBoVs was not significantly different in cases and controls.

HBoVs in the absence of other enteric pathogens were seen in 46% of the HBoV-positive samples, and more frequently in the controls, 50.3% vs 40.9% in cases. No significant difference in HBoV load was observed between cases and controls, or between the samples positive for HBoV alone or in the presence of other pathogens. Previous studies have investigated the relationships between viral load and disease severity [33], [34], [35], [36], [37]. In respiratory infections significantly higher HBoV loads were seen in samples collected from children positive for HBoV alone than in those from children with co-infections. In respiratory infections also, viral loads >10^4^ were associated with disease, whereas loads <10^4^ were associated with asymptomatic children [33], [38]. This lead to the suggestion that higher viral loads are indicative of a causative role of HBoV in respiratory infections [33], [38]. However, Brieu et al [38] found no significant correlation between viral load and clinical symptoms or disease severity.

In conclusion, the results obtained from investigating for the presence of HBoV DNA in archived DNA samples from a large and previously well described case-control study of IID suggest that HBoV, including HBoV-2,do not appear to be a significant cause of gastroenteritis in the UK population, and particularly in the paediatric population. Although HBoVs are relatively frequent across all ages, and in particular in preschool age children, they are found just as frequently among children and adults without symptoms of gastroenteritis.
